# Symbiosis between Cretaceous dinosaurs and feather-feeding beetles

**DOI:** 10.1073/pnas.2217872120

**Published:** 2023-04-17

**Authors:** Enrique Peñalver, David Peris, Sergio Álvarez-Parra, David A. Grimaldi, Antonio Arillo, Luis Chiappe, Xavier Delclòs, Luis Alcalá, José Luis Sanz, Mónica M. Solórzano-Kraemer, Ricardo Pérez-de la Fuente

**Affiliations:** ^a^Centro Nacional Instituto Geológico y Minero de España, Consejo Superior de Investigaciones Científicas, Valencia 46004, Spain; ^b^Departament de Dinàmica de la Terra i de l’Oceà, Facultat de Ciències de la Terra, Universitat de Barcelona, Barcelona 08028, Spain; ^c^Institut de Recerca de la Biodiversitat, Universitat de Barcelona, Barcelona 08028, Spain; ^d^Institut Botànic de Barcelona (CSIC-Ajuntament de Barcelona), Barcelona 08038, Spain; ^e^Division of Invertebrate Zoology, American Museum of Natural History, New York NY 10024-5192; ^f^Departamento de Biodiversidad, Ecología y Evolución, Facultad de Biología, Universidad Complutense, Madrid 28040, Spain; ^g^Dinosaur Institute, Natural History Museum of Los Angeles County, Los Angeles 90007; ^h^Parque de las Ciencias de Andalucía, Granada 18006, Spain; ^i^Unidad de Paleontología, Facultad de Ciencias, Universidad Autónoma de Madrid, Madrid 28049, Spain; ^j^Real Academia Española de Ciencias Exactas, Físicas y Naturales, Madrid 28004, Spain; ^k^Department of Palaeontology and Historical Geology, Senckenberg Research Institute, Frankfurt am Main 60325, Germany; ^l^Oxford University Museum of Natural History, Oxford OX1 3PW, UK

**Keywords:** arthropod-dinosaur interaction, symbiosis, amber, Cretaceous, paleoecology

## Abstract

Extant terrestrial vertebrates, including birds, have a panoply of symbiotic relationships with many insects and arachnids, such as parasitism or mutualism. Yet, identifying arthropod–vertebrate symbioses in the fossil record has been based largely on indirect evidence; findings of direct association between arthropod guests and dinosaur host remains are exceedingly scarce. Here, we present direct and indirect evidence demonstrating that beetle larvae fed on feathers from an undetermined theropod host (avian or nonavian) 105 million y ago. An exceptional amber assemblage is reported of larval molts (exuviae) intimately associated with plumulaceous feather and other remains, as well as three additional amber pieces preserving isolated conspecific exuviae. Samples were found in the roughly coeval Spanish amber deposits of El Soplao, San Just, and Peñacerrada I. Integration of the morphological, systematic, and taphonomic data shows that the beetle larval exuviae, belonging to three developmental stages, are most consistent with skin/hide beetles (family Dermestidae), an ecologically important group with extant keratophagous species that commonly inhabit bird and mammal nests. These findings show that a symbiotic relationship involving keratophagy comparable to that of beetles and birds in current ecosystems existed between their Early Cretaceous relatives.

## Significance

Vertebrates and arthropods are two of the most successful and frequently fossilized animal groups, but direct evidence of their interaction in deep time – entailing the joint, intimate fossilization of remains from both groups – is extremely rare. Our discoveries in fossilized plant resin (amber) from the Early Cretaceous of Spain show that a symbiotic relationship, likely commensal/mutualistic, was established between beetle larvae feeding on detached feathers and feathered dinosaurs (theropods) more than a hundred million years ago. Only two previous records of arthropod–theropod symbiosis involving direct fossil evidence were known, both parasitic. Our findings demonstrate that beetles and feathered theropods have interacted since the Mesozoic, and shed light on the evolutionary importance of early symbiotic relationships between arthropods and vertebrates.

Feathers, like hair, are integumentary structures composed of tough, durable keratin. Despite being a concentrated source of this protein, few groups of arthropods have evolved adaptations to ingest and metabolize keratin (keratophagy) (1, 2). Aside from its ecological significance, keratophagy is important from an evolutionary standpoint as well, representing, for example, a transitional stage between free-living bark lice (Psocodea) and true parasitic lice (Phthiraptera), in the form of scavenging book lice (genus *Liposcelis*) that commonly feed on nest debris, including keratin (1). Keratophagy as a trophic specialization entails a parasitic symbiosis if the feeding arthropod guest causes damage in the integument of the vertebrate host (3). On the contrary, keratophagy can also involve a commensal–mutualistic symbiosis between the host and the arthropod consuming the host’s shed, accumulated integumentary structures, possibly advantageous to the host by cleaning its nest (4). In any case, symbiotic interactions (used herein in the wide sense) often do not fit into one of the traditional categories (3). Behaviors involving keratophagy do not necessarily represent trophic specializations, such as reptiles eating shed skins from themselves or conspecifics (5).

The plumage of diverse theropods has been well characterized in finely preserved Mesozoic compression fossils (6, 7). In Cretaceous amber, theropods are much less diverse, but the feathers are preserved in unmatched detail (8–13). In contrast, the only definitive keratophagous arthropod hitherto identified in the fossil record is a chewing louse (Amblycera) of the family Menoponidae, preserved as a compression fossil from the Eocene of the German outcrop of Messel; the remains of feather barbules preserved in its gut are direct evidence of keratophagy (14). Moreover, only a few Mesozoic (15, 16) and Cenozoic (17–20) amber records are known to contain arthropods associated with remains of the vertebrate host (feathers and hair), indicating symbiotic relationships. These Mesozoic amber records represent direct evidence of an arthropod–dinosaur symbiotic relationship, all entailing instances of ectoparasitism (15, 16). A few Mesozoic records of arthropods associated with remains of the vertebrate host also exist in compression strata (21, 22); yet due to preservational and taphonomic limitations in lithological matrix, the interpretation of these assemblages is usually ambiguous.

A recent, controversial report involves minute, wingless insects preserved adjacent to feathers in Burmese amber, reported as keratophagous ectoparasites, and placed in a new family, Mesophthiridae (23). Reassessment of the morphology, however, indicated that these are actually early instars (crawlers) of scale insects (Coccoidea), a group that today is wholly phytophagous, siphoning plant vascular fluids using long, very fine stylets (24). These stylets are coiled internally when at rest, forming a very distinctive structure called the crumena, which is clearly visible in the images from the original report along with other features diagnostic of coccoids. It was proposed that these fossil coccoids may have been merely phoretic, but they definitely were not feeding on the feathers (24, 25), despite some feather portions with apparent feeding damage, probably produced by other arthropods. In a rebuttal by the original authors, new images still did not resolve the putative chewing mandibles (26). The morphological evidence is unequivocal that Mesophthiridae are coccoids and thus could not have been keratophagous.

Here, we present assorted evidence of keratophagy involving beetle and feather remains preserved in Cretaceous amber from Spain, representing a rare instance of arthropod–dinosaur symbiotic relationship in deep time.

## Results

### Amber Samples Studied.

**Fig. 1.**
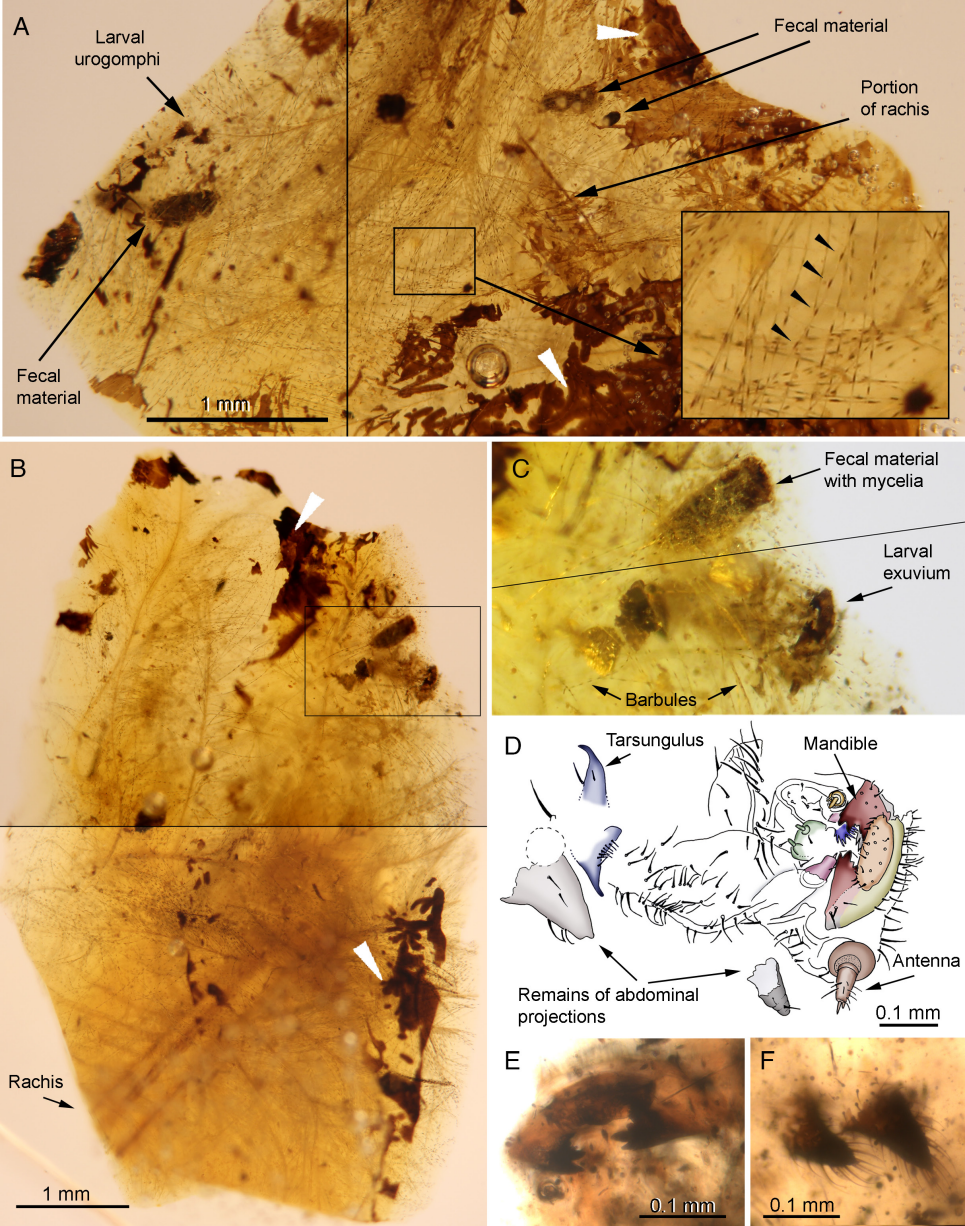
Exuvial remains from keratophagous beetle larvae intimately associated with plumulaceous feather remains in amber preparation of SJNB2012-31-01 from the San Just outcrop (NE Spain), upper Albian (Early Cretaceous) in age. (*A*) Amber fragment (F3, see *SI Appendix, *Fig. S1) with feather and exuvial remains, showing detail of the barbule node pigmentation (inset arrowheads). (*B*) Amber fragment (F2, see *SI Appendix, *Fig. S1) with partial plumulaceous feather and exuvial remains. (*C*) Detail of the largely intact larval exuvium in ventral view, and fecal material with fungal growth (mycelia; inset in *B*), both surrounded by barbules. (*D*) Larval exuvium in ventral view. (*E*) Larval head in frontal view showing mandibles. (*F*) Isolated pair of urogomphi (see *A* for location). Note the dark desiccation surfaces typical of aerial amber in *A* and *B* (white arrowheads). Images (*A*–*C*) composed of photographs taken at different focal planes. Black lines in some photos delineate the edges of tiled photographs.

**Fig. 2.**
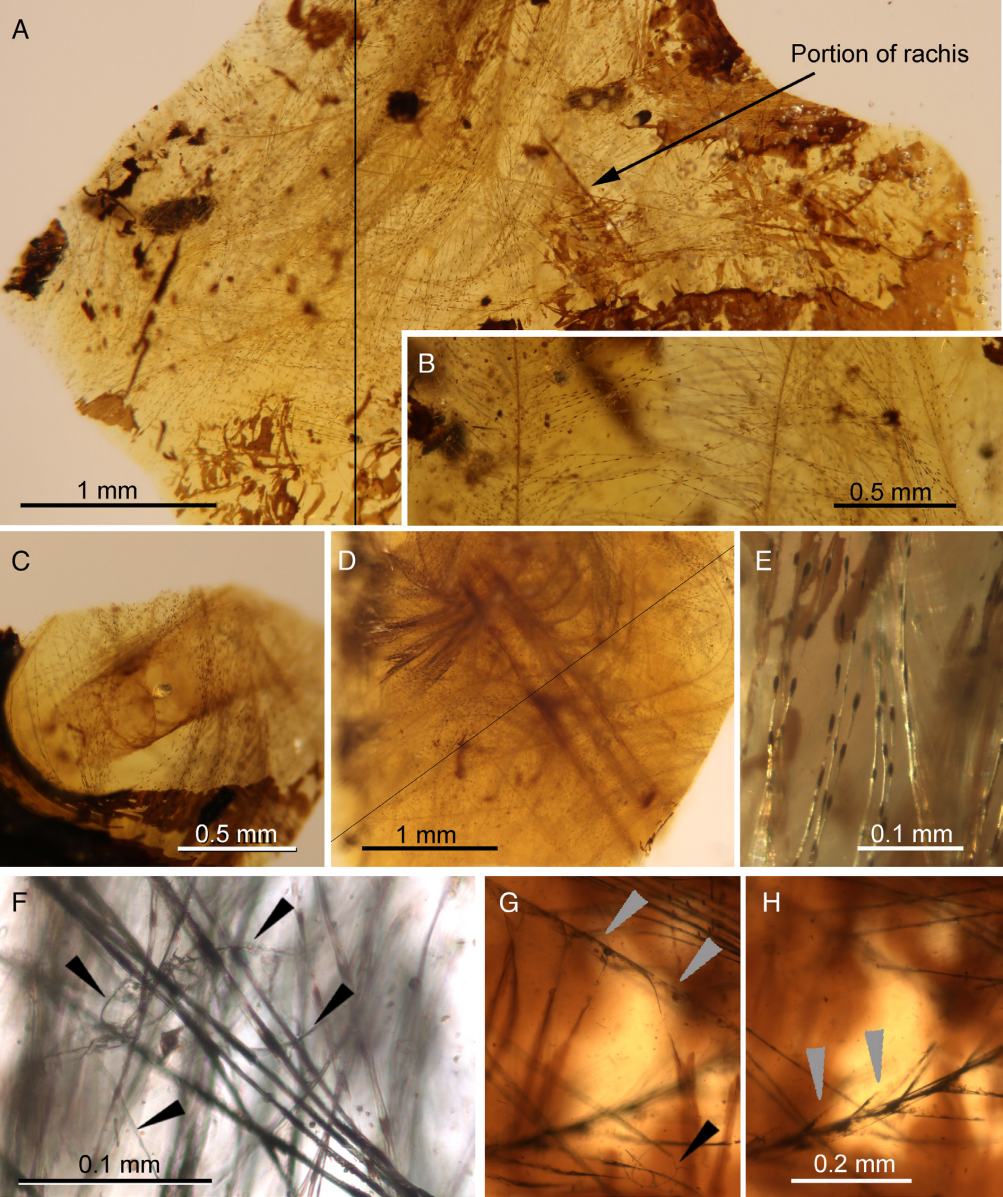
Morphological features and barb degradation of the feather portions associated with exuvial remains of beetle larvae in amber preparation of SJNB2012-31-01 from the San Just outcrop (NE Spain), upper Albian (Early Cretaceous) in age. (*A*) Feather remains showing a fragment of rachis and abundant barbs. (*B*) Two conspicuous barbs with abundant barbules. (*C*) Calamus and abundant surrounding barbs. (*D*) Portion of rachis showing abundant barbs arising from it. (*E*) Detail of the brown pigmentation concentrated in nodes of barbules. (*F*) Hyphae (black arrowheads) entangled or growing on the barbules. (*G* and *H*) Barb degradation showing hyphae on the barbules (black arrowhead) and barbule incompleteness (gray arrowheads) (both images of the same area at different focal planes and at the same scale). (*A* and *E*) from amber fragment F3; (*B* and *D*) from F2; (*C*) from F4. Note the dark desiccation surfaces typical of aerial amber in *A, C,* and *E*. Images (*A* and *D*) composed of photographs taken at different focal planes.

The amber piece SJNB2012-31 (Figs. 1 and 2 and *SI Appendix*, Figs. S1–S3), from the San Just outcrop (upper Albian, NE Spain), was separated into two preparations, the principal one (SJNB2012-31-01) containing five amber fragments (largest about 8 × 6 mm). Altogether, the amber fragments contain abundant feather remains, exuvial (molt) remains of beetle larvae, fecal material, and debris. Some amber fragments show desiccation surfaces typical of above-ground (aerial) resin (Fig. 1 and *SI Appendix*, Figs. S1 and S3). The feather remains include abundant barbs of plumulaceous feather portions, an incomplete calamus (1.15 mm long as preserved, 0.40 mm greatest width), and two segments of rachises (0.4 mm wide), showing a rachidial ridge (Fig. 2). Plumulaceous barbs (up to 5.6 mm long as preserved) show brown pigmentation in barbule nodes. Exuvial remains in preparation SJNB2012-31-01 include a rather complete exuvium (Fig. 1 *B*–*E*) and other more fragmentary remains; all of these are associated with a cloud of barbules (*SI Appendix*, Figs. S1 and S3 and Movie S1) and were shed by at least two conspecific larval individuals of minute size (0.61 mm long as preserved, larvae estimated to have been ~1.5 mm) and of an early developmental stage. The six distinct exuvial remains preserved in different amber fragments (F#) of the preparation containing the feather–beetle assemblage (SJNB2012-31-01 and *SI Appendix*, Fig. S1) are: 1) a rather intact exuvium lacking urogomphi due to disarticulation, in F2 (Fig. 1 *B* and *C* and *SI Appendix*, Fig. S2); 2) a fragment of a degraded abdominal projection, in F2; 3) a prementum portion, in F3 (*SI Appendix*, Fig. S2*D*); 4) a highly decayed fragment of an abdominal projection, in F3 (*SI Appendix*, Fig. S3*B*); 5) a fragment of a thoracic leg fragment with tarsungulus, in F4 (*SI Appendix*, Fig. S3*A*); and 6) paired urogomphi, in F3 (Fig. 1*A* and  *SI Appendix*, Fig. S3*C*). In addition, minute cuticle remains that may belong to degraded exuviae of this type are also present. The fecal material corresponds to several barrel-shaped structures (ca. 0.36 × 0.14 mm), most of them with fungal mycelia (~1 µm thick) growing on them.

**Fig. 3.**
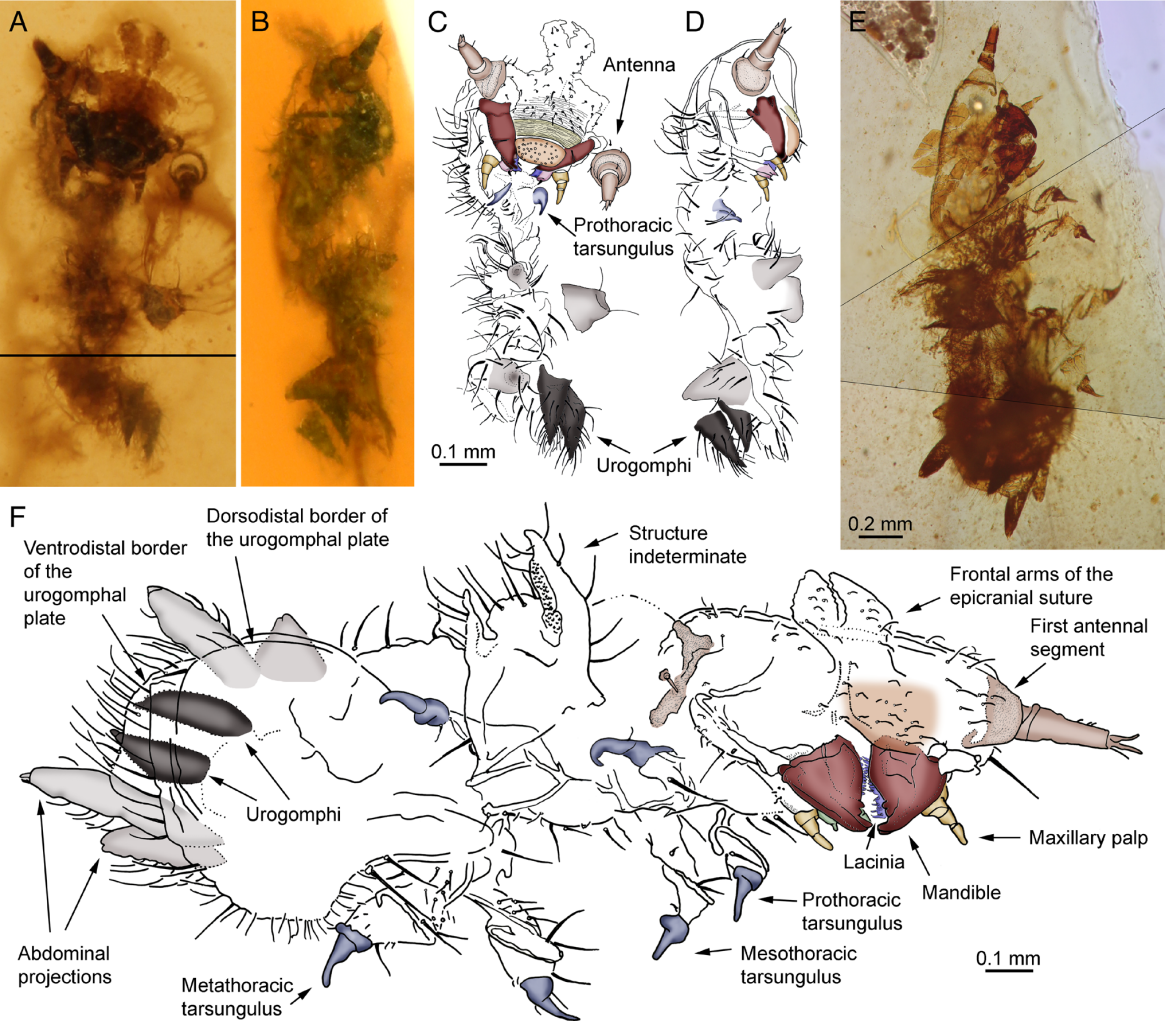
Isolated exuvium in amber piece SJNB2012-11 from the San Just outcrop (NE Spain), upper Albian (Early Cretaceous) in age (*A*–*D*), and isolated exuvium belonging to a putatively conspecific, more advanced instar from El Soplao amber (N Spain), middle Albian in age, piece ES-07-39 (*E* and *F*). (*A* and *B*) Ventral and lateral views, respectively. (*C* and *D*) Drawings of the former views. (*E*) Ventral oblique view. (*F*) Drawing of the same exuvium. (*A*–*D* and *F*) at the same scale for comparison. Images (*A* and *E*) composed of photographs taken at different focal planes.

Three isolated, conspecific exuviae, two of them virtually complete, are preserved in different amber pieces and allow a more detailed account of the beetle larva studied herein. The first isolated exuvium (SJNB2012-11: 0.83 mm body length) is an early developmental stage as the exuviae in SJNB2012-31, and belongs to the same amber-bearing stratum in San Just (Fig. 3 *A*–*D* and Movie S1). The amber piece with the inclusion is a clear amber fragment lacking any other biological inclusions or desiccation surfaces. The second isolated larval exuvium, in piece ES-07-39, was found in the slightly older El Soplao outcrop (middle Albian, N Spain) (Fig. 3 *E* and *F* and *SI Appendix*, Figs. S4 and S5). The exuvium is very intact and well preserved (only one antenna is lost, clypeus and labrum are largely obscured). It shows the three pairs of thoracic legs and the complete posterior body portion in excellent detail and in original position. Although about two times larger and with a mandibular prostheca apparently double rather than simple (*SI Appendix*, Fig. S4), this exuvium shows virtually the same morphological characters as those from the exuvial remains from San Just, and so it most likely represents a conspecific, later instar. The amber piece lacks any other biological inclusions or desiccation surfaces. The third exuvial specimen, in piece MCNA 12063, is partial and consists of a posterior body portion exceptionally preserved, and was found in the Peñacerrada I outcrop (upper Albian, N. Spain) (*SI Appendix*, Fig. S6). As the preserved body portion is significantly larger than the corresponding part from the El Soplao specimen and the visible features are consistent with those shown by the other exuviae, it could represent an even later instar of a conspecific larva.

Although the exuviae from San Just represent the earliest developmental stage of the larva studied herein, they are the most palaeoecologically significant remains due to their association with feather portions. For that reason, the descriptive account provided below focuses on these exuviae, with comparative notes on the increasingly more advanced larval instars found in El Soplao and Peñacerrada I localities.

### Description of the Larval Exuviae.

Exuviae produced by early instar larvae (SJNB2012-11; SJNB2012-31). Setation abundant, dense (particularly on the thorax and abdomen), relatively long (ca. 10–20 µm); setation simple, without fine feathering, scales or plumosity. Cephalic capsule orthognathous, probably slightly more declined, without visible stemmata, with setae distinctly shorter. Cephalic setal arrangement not discernible due to cuticular crumpling. Frontal arms of epicranial suture lyriform, likely contiguous at base (Fig. 3 *A* and *C* and *SI Appendix*, Fig. S2*A*). Antenna (*SI Appendix*, Figs. S2 *E* and *F*) relatively short, laterally inserted (this feature likely impacted by preservation), ~10 µm length, well sclerotized, three segmented. First antennal segment (putative scape) ring-like, diameter ca. 1.5 times that of second antennal segment, length half that of the second antennal segment. Second antennal segment (putative pedicel) subcylindrical, slightly tapering distad, about 1.7 times longer than wide at base, bearing abundant fine setae and two thicker setae emerging from the subapical margin of the segment, these setae about as long as half the length of the segment; sensorium emerging apically from second antennal segment, small, conical, almost or as long as third antennal segment; one strong seta also emerging from the apex of the second antennal segment. Third antennal segment (putative flagellum) spine-like, lacking a terminal seta. Clypeus with fine setae, slightly broader but shorter than labrum; frontoclypeal suture evident. Labrum free, approximately trapezoidal in shape, without subdivisions, wider than long, with short setae; setae on margin near mouth thicker. Mandibles (Figs. 1*E* and 3 and *SI Appendix*, Fig. S2*C*) bidentate (a minute protrusion on the external margin is not considered a third mandibular tooth apparently smaller due to perspective), symmetrical; teeth subequal (inner tooth in the left mandible slightly larger in appearance probably due to perspective), innermost tooth basally expanded at incisor edge; prostheca present, as a single unsclerotized process, lacking visible setae; molar area present, visible surface crenulated, oblique (at least distally), vertical portion (if present) not visible (in left mandible); base of mandibles with some fine, short setae. Maxilla (*SI Appendix*, Fig. S2*B*) with stipes well developed, longer than wide, slightly sclerotized, separation with cardo unrecognizable; maxillary palp three segmented, with distinct palpifer, segment lengths 3>2>1; diameter of basal palpomere about twice that of apical one; apical palpomere with minute, clavate setula laterally; galea and lacinia separated; inner margin of lacinia spinose. Galea bearing uncus. Labium with prementum (*SI Appendix*, Fig. S2 *A* and *D*) well developed, roughly quadrate, ligula absent; labial palp two segmented, well separated from each other. Presence/absence of gula not visible. Tarsunguli well developed and sclerotized, simple, claw-like, with distal half tapering, setae absent (*SI Appendix*, Fig. S3*A*). Other thoracic leg parts present, disarticulated proximally and/or distally (truncated in shape). One pair of urogomphi evident, simple, unsegmented, relatively short, triangular, laterally flattened; slightly tapering distally, with minute spine at the apex (Fig. 1*F* and *SI Appendix*, Fig. S2*H*); urogomphi in the dorsal position and posteroventrally curved (Fig. 3*D*), slightly inclined toward each other (not upturned), covered with dense, fine setae. Additional abdominal structures (herein referred as “abdominal projections”) preserved as remains of their apices, but their exact configuration is unclear.

The exuvium from El Soplao (ES-07-39), produced by an older instar, has the distal portion of the abdomen more fully preserved than in the earlier instar exuviae from San Just. The specimen has three visible paired abdominal structures, the distalmost interpreted as a pair of urogomphi, medially placed on a plate in segment IX, and two pairs of projections in more lateral position, on segments VII and VIII (*SI Appendix*, Fig. S5). Although the urogomphi are in an apparent ventral position, they were likely displaced during molting, sharing the features of the above-described urogomphi from the San Just exuviae. The distalmost pair of lateral abdominal projections are longer and conical in shape, bearing a few fine setae and with a rounded apex; the more proximal pair of projections are shorter and apparently more flattened in shape, bearing a few thick setae and having a notched apex. The preserved abdominal projections from the San Just specimens are compatible with those of ES-07-39, although it is unknown whether the abdominal configurations of the two forms matched. Yet, it is probable that the abdominal projections were less developed in earlier developmental stages.

Lastly, the partial exuvial specimen from Peñacerrada I (MCNA 12063), produced by a late instar, shows in the distalmost pair of lateral abdominal projections an inner, central tube with the surface finely striated (*SI Appendix*, Fig. S6). This structure is deemed as part of the tracheal system ending in a (putatively spiracular) opening at the apex of the projection. Moreover, the specimen shows additional lateral projections on more proximal abdominal segments.

## Discussion

### Affinities of the Larvae.

The morphology of the described larval exuviae, including those associated with feather remains, firmly places them as beetle (Coleoptera) larvae. The more intact exuviae show powerful chewing mandibles with highly sclerotized teeth, and with a pair of dorsal abdominal processes identified as urogomphi that are slightly recurved and cone shaped. This type of urogomphus is typical of active, campodeiform beetle larvae inhabiting narrow spaces as it assists in locomotion (27). The character combination of the fossils is most consistent with that in extant representatives of the families Dermestidae and Derodontidae, namely in characters of the head and its appendages (*SI Appendix*, Table S1). The orthognathous head in the three more intact exuviae (both early and more advanced instars) is present in dermestid larvae and distinguishes them from other potential beetle groups to which they could be related based on morphology. Some mandibular characters present in the new fossils occur scattered in larvae of extant genera and other groupings of Dermestidae: The mandible is apically bidentate in *Orphilus*, Thorictinae, and most Trinodinae; the prostheca is variable, sometimes a blunt process, but not sclerotized; and although a mola is usually absent, it is present in *Orphilus* (28). In contrast, although the frontal arms of the epicranial suture in extant dermestid larvae are V- or U-shaped (29), they are lyriform in the new fossils. The abdominal projections from the fossil exuviae, lateral in position as shown by the more advanced instar larvae, are absent from the known extant diversity of Dermestidae (28–31). Note that, although specialized setae such as spicisetae or hastisetae are absent in *Orphilus*, some dermestid species possess short, undulating (“bent”) setae (30, 31); the absence of all these setae can be confirmed in the fossils.

Derodontids have been considered either sister to dermestids (28) or relatively close phylogenetically yet classified in a different infraorder (27). However, recent phylogenomic studies consistently recover Derodontidae as phylogenetically distant from Dermestidae, sister to Clambidae and Eucinetidae (32–34). Although the frontal arms of the epicranial suture are lyriform in extant derodontid larvae, as in the fossil exuviae, their head is prognathous and their mandibles possess a falciform prostheca, both characters absent in the fossils.

The affiliation of these fossilized larval remains based on their morphology to yet other coleopteran lineages, such as Jacobsoniidae – currently classified in Staphyliniformia sensu Cai *et al*. (34) – or several cucujiformian families remains a possibility, although a less likely one based on the number of shared characters (*SI Appendix*, Table S1).

### Taphonomy.

Within the amber assemblage preserved in the preparation of SJNB2012-31-01, the exuvial remains are fully enveloped by the plumulaceous feather portions (Fig. 1 and *SI Appendix*, Fig. S3). Based on such an intimate association, as well as the rarity of feathers and these exuviae in amber, it is highly improbable that both kinds of inclusions were independently trapped in resin and, thus, accidentally associated. Moreover, several elements in the amber fragments of the preparation show substantial degradation. The two more intact exuviae lack some body parts, and several fragmentary exuvial portions are present. Some fungal hyphae are present on feather barbules, the latter also showing degradation in some areas (Fig. 2 and *SI Appendix*, Fig. S3). Lastly, the four coprolites were probably produced by the beetle larvae prior to molting and fungal hyphae subsequently grew on them (*SI Appendix*, Fig. S3). Therefore, the exuvial and feather remains were most likely associated for a period of time prior to becoming jointly immersed in resin.

Evidence of extensive feeding damage (chewing) is absent in the feather portions surrounding the exuvial remains, but localized barbule damage is present in a few areas (*SI Appendix*, Fig. S2 *G* and *H*), although this degradation might be at least partly preservational. The tridimensional branching of the plumulaceous feather portions (not bidimensional as in pennaceous feathers) and their twisted configuration might also prevent the identification of more evident feeding damage, as for instance present in the feather figured by Gao *et al*. (23) in their figure 1 *A* and *K*.

### Keratophagy.

The most compelling interpretation of the fossil exuviae, based on the preserved morphological, systematic, and taphonomic data, is that they are remains of keratinophagous, dermestid (in the wide sense) beetle larvae that retained a few plesiomorphic characters, such as lyriform frontal arms of the epicranial suture, while also possessing apparently derived lateral abdominal processes. The minute to small size of the fossil exuviae is in line with keratin feeding, as this protein has a poor nutritional value.

**Fig. 4.**
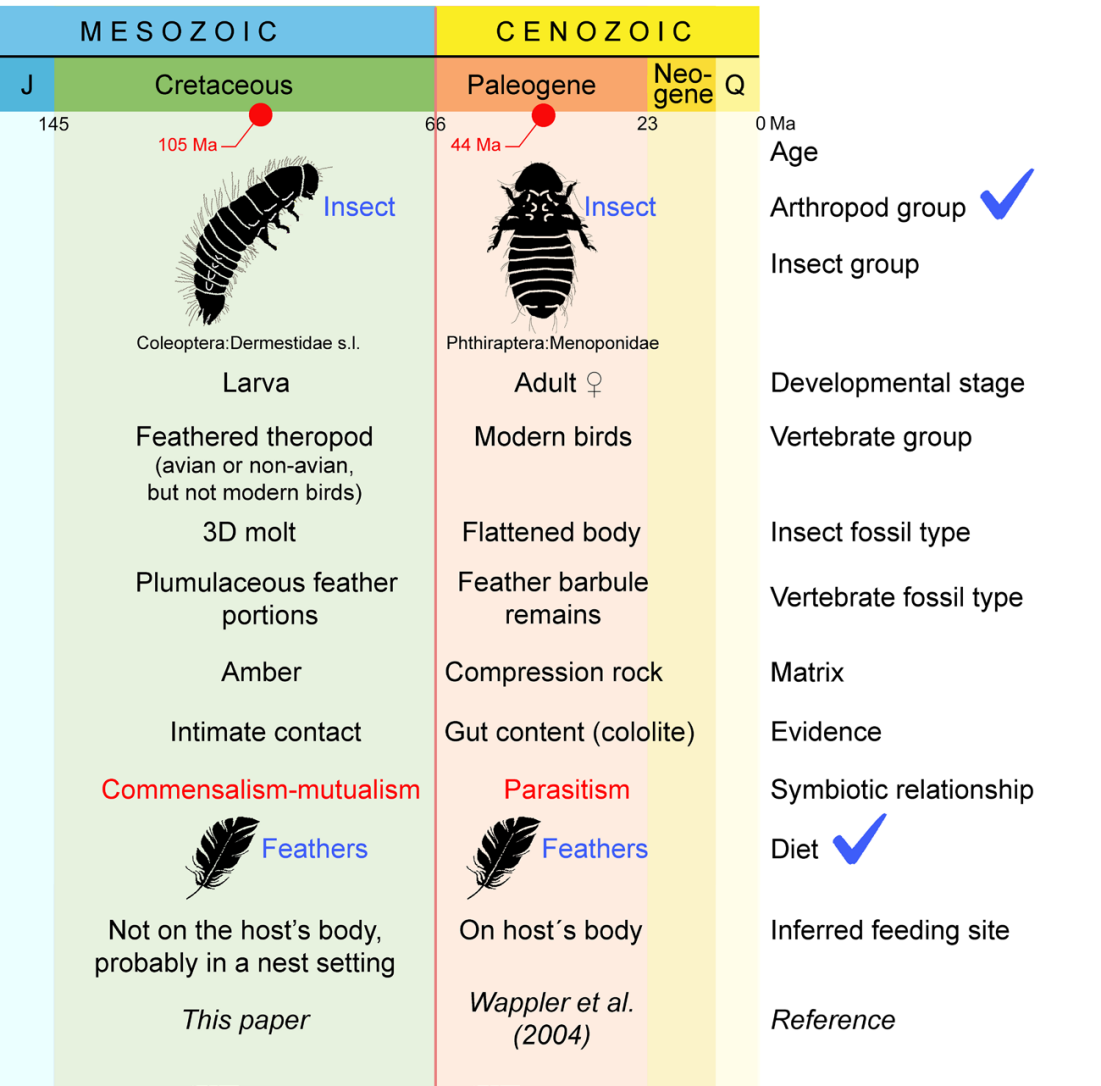
Comparison of the only two records providing direct evidence of keratophagy in the current fossil record. The most relevant difference between the two records from the evolutionary standpoint is the type of symbiotic relationship recorded (in red). Only two important features are shared between the records, which are the arthropod group involved and integumentary food consumed (in blue). Abbreviations: J = Jurassic, Q = Quaternary.

There are approximately 1,700 extant species of dermestid beetles (35), and the family is nearly global. The Mesozoic amber record of both adults and larvae is well represented in deposits worldwide. This includes some adults from Cretaceous ambers, both described (36) and undescribed (37, 38), as well as larvae, the latter as body records (1, 39) (Fig. 4 *E*–*G*) or as hastisetae, which are very diagnostic for certain subfamilies (15, 40, 41). Extant dermestids, especially larvae, are xerophilic scavengers of animal materials, particularly dried matter including keratin-based tissues (29). These beetles are one of the most important pests of stored products and dried museum collections, and are also commonly found in extant vertebrate nests, with the latter representing the ancestral habitats of many keratophagous insects (29, 42–44). Since extant dermestid beetles are typical inhabitants of bird nests (42, 43), and the assemblage reported herein is comprised of dermestid larvae and feather remains preserved together, the most plausible microenvironment where it originated was a nest, where feathers accumulate and sustain populations of the beetles. As for many families of beetles, the larval stages of dermestids live longer than adults and are the primary feeding stage.

Extant bird and mammal nests are specialized microenvironments and rich sources of organic material inhabited by a diverse community of insects and arachnids (42, 43, 45). Many of these nest-inhabiting arthropods are generalist or specialist feeders on the organic remains, including shed keratin in the form of feathers, hair, and skin, as well as feces. Extinct deinocrotonid ticks preserved in ca. 100-million-y-old Burmese amber, some associated with feathers, are considered to have been nidicolous − living in their host’s nest or in their own nest close to that of the host − with dermestid hastisetae attached to their bodies and other taphonomic features linking these hematophagous ectoparasites to a nest microenvironment (15). It is unlikely that beetle larvae were feeding on detached feathers outside of a nest, since feathers would have been dispersed, and nest associates breed where the foodstuff is concentrated (45). On the contrary, it is also unlikely that the feathers were still attached to its host when the larvae fed on them due to the fungal hyphae growing on barbules and the coprolites, indicating some decay.

Keratophagous arthropods are obligate symbionts that depend on their hosts as reliable sources of food and shelter. Due to generalized morphology of these Early Cretaceous plumulaceous feathers, it is not possible to determine the group of Cretaceous feathered theropod (avian or nonavian) to which they belonged. However, modern birds (Neornithes) can be ruled out since they appeared later in the fossil record, during the latest Cretaceous based on fossil evidence (6). Also, the exact type of symbiotic relationship between the keratophagous beetle larvae and its feathered theropod host is challenging to address, even for extant fauna, given the mutualism–commensalism–parasitism spectrum (3, 46). A harmful impact (i.e., skewing toward parasitism) of the fossil dermestids to the feathered host was unlikely since they appear to have fed on detached feathers, and the larvae lacked hastisetae or spicisetae. These specialized setae, present in some dermestid taxa, easily detach and can accumulate so densely in nests as to form mats; the setae entangle ants and other predators (47) but can also irritate the nest hosts, in severe cases leading to death (48).

Both the Eocene chewing louse from Messel preserved with feather fragments in its gut (14) and the assemblage described herein represent insect–theropod interactions but have significant differences (Fig. 4). The most remarkable of them is that the Eocene record involved modern birds and entailed a parasitic symbiotic relationship provided that chewing lice cause damage to the feathers while these are attached to the host, not as scavengers, thus impacting on the bird’s fitness. On the contrary, our record involved detached feathers from a Cretaceous feathered theropod (avian or nonavian), thus most likely representing a commensal or mutualistic symbiotic relationship (Fig. 4).

## Concluding Remarks

The present findings provide direct and indirect evidence of early insect keratophagy in a commensal or mutualistic relationship between arthropods and dinosaurs during the late Mesozoic. Taphonomically, it is most likely that the beetle larvae that produced the exuviae fed on accumulated feathers in or very near a resin-producing tree, probably in a nest setting. Previous fossil records of arthropod–theropod relationships based on direct evidence have been of parasites, either of chewing lice (keratophagy) during the Eocene or of ixodid and deinocrotonid ticks (hematophagy) during the Cretaceous. The emerging view is that some groups of arthropod symbionts of feathered theropods in the late Mesozoic transitioned to modern birds in the Cenozoic, an observation made possible by preservation with unique fidelity in amber.

## Materials and Methods

### Amber Pieces.

The four amber pieces come from three Spanish outcrops. Two of them were excavated from a level rich in amber and charcoal within the San Just outcrop (49, 50), upper Albian in age, during a paleontological excavation carried out in 2012 (Gobierno de Aragón permit: 119/10-11-2012). This outcrop is close to the village of Utrillas, Teruel Province (Autonomous Community of Aragón). Preparation of SJNB2012-31-01 (Figs. 1 and 2 and *SI Appendix*, Figs. S1, S2, and S3) contains five small amber fragments (F1 to F5, ranging from 2 to 7 mm in length) originally belonging to the same amber piece which fragmented during preparation, and which were embedded and polished in the same prism of synthetic resin (EPO-TEK 301; 24 × 16 × 4 mm in size) (51). An additional isolated fragment belonging to the same amber piece (F6; accession number SJNB2012-31-02) was prepared separately and contains a wasp. Exuvial remains have a slightly fragmented and shriveled appearance. In total, this piece contains an incomplete beetle larva exuvium and four small, isolated, conspecific exuvial portions at least one of them belonging to a different individual. Piece SJNB2012-11 (Fig. 3 *A*–*D*; *SI Appendix*, Fig. S2) contains an isolated, conspecific, almost complete beetle exuvium consisting of an amber portion (8 × 6 × 1 mm) embedded and polished in the same type of synthetic resin prism (20 × 14 × 1 mm in size). Both pieces are housed in the collection of the Museo Aragonés de Paleontología (Fundación Conjunto Paleontológico de Teruel-Dinópolis, Teruel city). The third amber piece, ES-07-39 (Fig. 3 *E* and *F* and *SI Appendix*, Figs. S4 and S5), was excavated from a rich level within El Soplao outcrop (Rábago village, Santander, Cantabria) (52, 53), middle Albian in age, during a paleontological excavation carried out in 2007 with a permit of the Gobierno de Cantabria. It contains a virtually complete beetle exuvium consisting of an amber portion (5 × 3 × 1 mm) embedded and polished in the same type of synthetic resin prism (23 × 10 × 2 mm in size). The piece is housed at the Institutional Collection from the El Soplao Cave, Cantabria, Spain. The fourth amber piece, MCNA 12063 (*SI Appendix*, Fig. S6), was excavated by researchers of the Museo de Ciencias Naturales de Álava, in Peñacerrada I outcrop (Álava, Basque Country) (54), upper Albian in age (55). It contains a beetle exuvial portion, a rhagionid fly, a tipuloid fly, an indeterminate wasp, a fragment of a termite wing, arthropod coprolites, and diverse minute debris, present in an amber portion (15 × 10 × 3 mm) embedded and polished in the same type of synthetic resin prism (20 × 15 × 4 mm in size). The piece is housed at the Museo de Ciencias Naturales de Álava, a Spanish public scientific institution.

### Imaging.

The specimens were examined with both Olympus BX51 and BX53 compound microscopes and the drawings were made using an Olympus U-DA drawing tube (camera lucida) attached to both the compound microscopes at the Instituto Geológico y Minero de España (CN IGME-CSIC, Madrid, and Valencia). Larval exuviae from San Just amber were described based on observation at 400× using a Nikon Eclipse compound microscope with Plan Apo Extended Depth Working Distance lens at the AMNH (New York). Photomicrographs were made using a digital camera attached to both Olympus compound microscopes at the CN IGME-CSIC; a movie sequence using selected images is included in Movie S1.

Confocal microscopy was used to image the exuvium in contact with feathers using a Leica TCS SPE-DM 5500 CSQ V-Vis (Manheim, D-68165, Germany) at the Museo Nacional de Ciencias Naturales (CSIC, Madrid). Images were acquired with a solid-state laser operating at 488 nm, a 10× eyepiece, an ACS APO 10×/0.3 objective, and the Leica Application Suite Advanced Fluorescence software (Leica MM AF 1.4). Fluorescence emission was collected from approximately 10 nm above the excitation wavelength up to 800 nm. Laser power for acquisition was set by viewing the fluorescence emission and increasing the power until the rate of increase in fluorescence slowed down. The photomultiplier gain for acquisition was then set by viewing the image and increasing the gain until signal overload was detected, at which point the gain was reduced slightly. Pixel matrices of 2048 × 2048, speed of 400 Hz, and frame average of 4 were acquired for each Z-step at a zoom setting of 1.5 to 2×. An Airy unit setting of 1 was routinely used for the observation pinhole. No attempts were made to optimize image quality by minimization of the confocal pinhole diameter; however, high levels of signal averaging, high pixel resolution, and very small Z axis steps were used. A movie sequence using selected confocal microscopy images is included in Movie S1.

## Supplementary Material

Appendix 01 (PDF)

Movie S1. Confocal laser scanning microscopy images showing a beetle exuvium among feather portions and optical microscopy images of the head from a conspecific isolated exuvium from the same stratigraphic level in upper Albian San Just amber outcrop. Preparation SJNB2012-31-01 (amber fragment F2) and piece SJNB2012-11. Both specimens viewed in frontal view. Stacking slideshows are based on photographs taken at successive focal planes.

## Data Availability

All study data are included in the article and/or *SI Appendix*.
