# Supplementary material for: Symbiosis between Cretaceous dinosaurs and feather-feeding beetles
Source: Proc Natl Acad Sci U S A. 2023 Apr 17;120(17):e2217872120. doi: 10.1073/pnas.2217872120 (PMC10151472; doi:10.1073/pnas.2217872120)
Supplement: Supplementary file 1 — Appendix 01 (PDF) [file pnas.2217872120.sapp.pdf]

PNAS

Supporting Information for

Symbiosis between Cretaceous dinosaurs and feather-feeding beetles

Enrique Peñalver, David Peris, Sergio Álvarez-Parra, David A. Grimaldi, Antonio Arillo, Luis Chiappe, Xavier Delclòs, Luis Alcalá, José Luis Sanz, Mónica M. Solórzano-Kraemer and Ricardo Pérez-de la Fuente

Corresponding authors: Enrique Peñalver and Ricardo Pérez-de la Fuente
Email: e.penalver@igme.es, ricardo.perez-de-lafuente@oum.ox.ac.uk

This PDF file includes:

Figures S1 to S6
Table S1
Legend for Movie S1
SI References

Other supporting materials for this manuscript include the following:

Movie S1

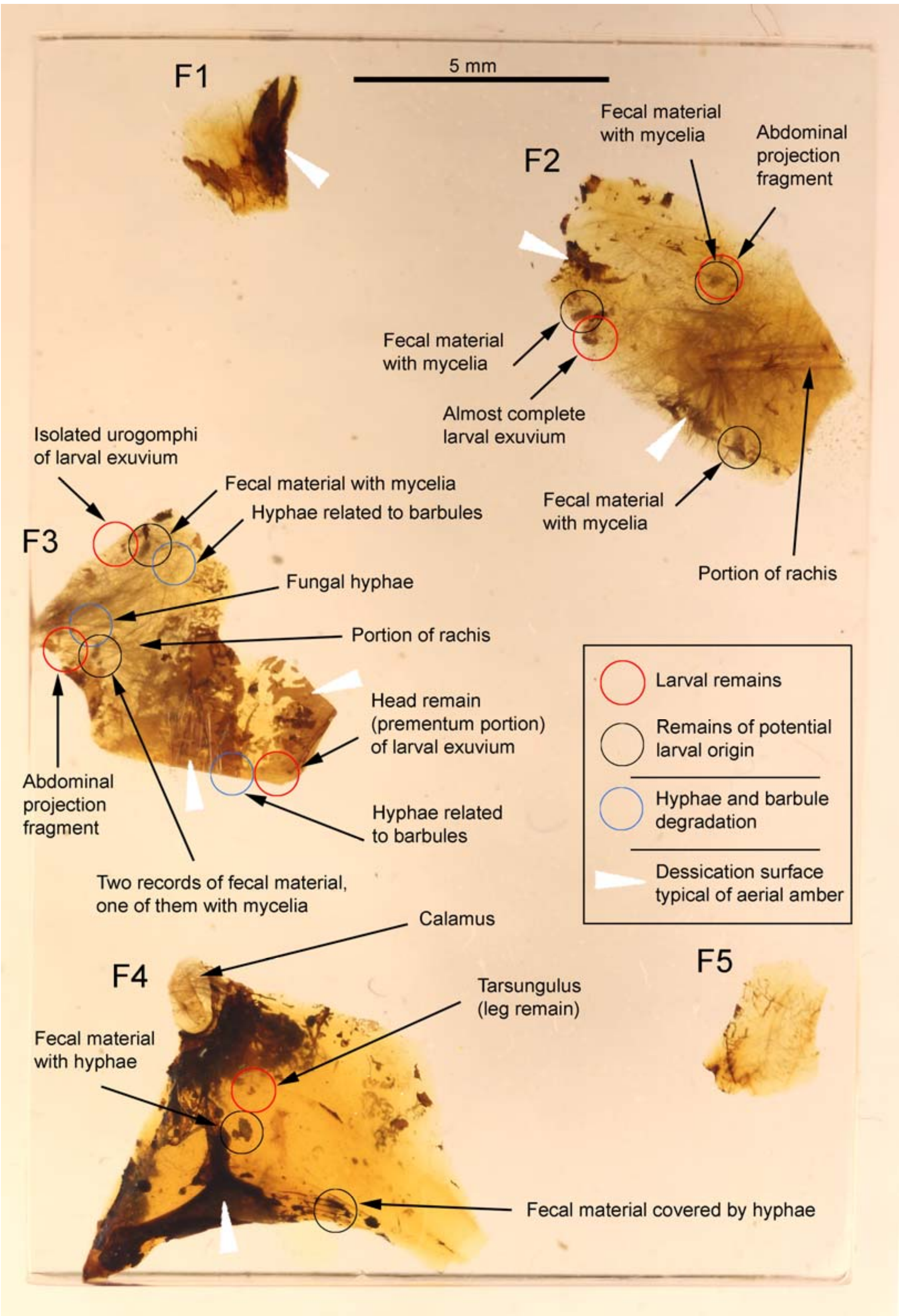

Fig. S1. Epoxy resin preparation (SJNB2012-31-01) bearing the fossil assemblage represented by five fragments (F1–F5) containing feather and beetle larvae exuvial remains. These fragments originally belonged to the same Spanish amber piece (Piece SJNB2012-31), San Just outcrop, upper Albian in age, which broke during preparation. Structures of interest are tagged.

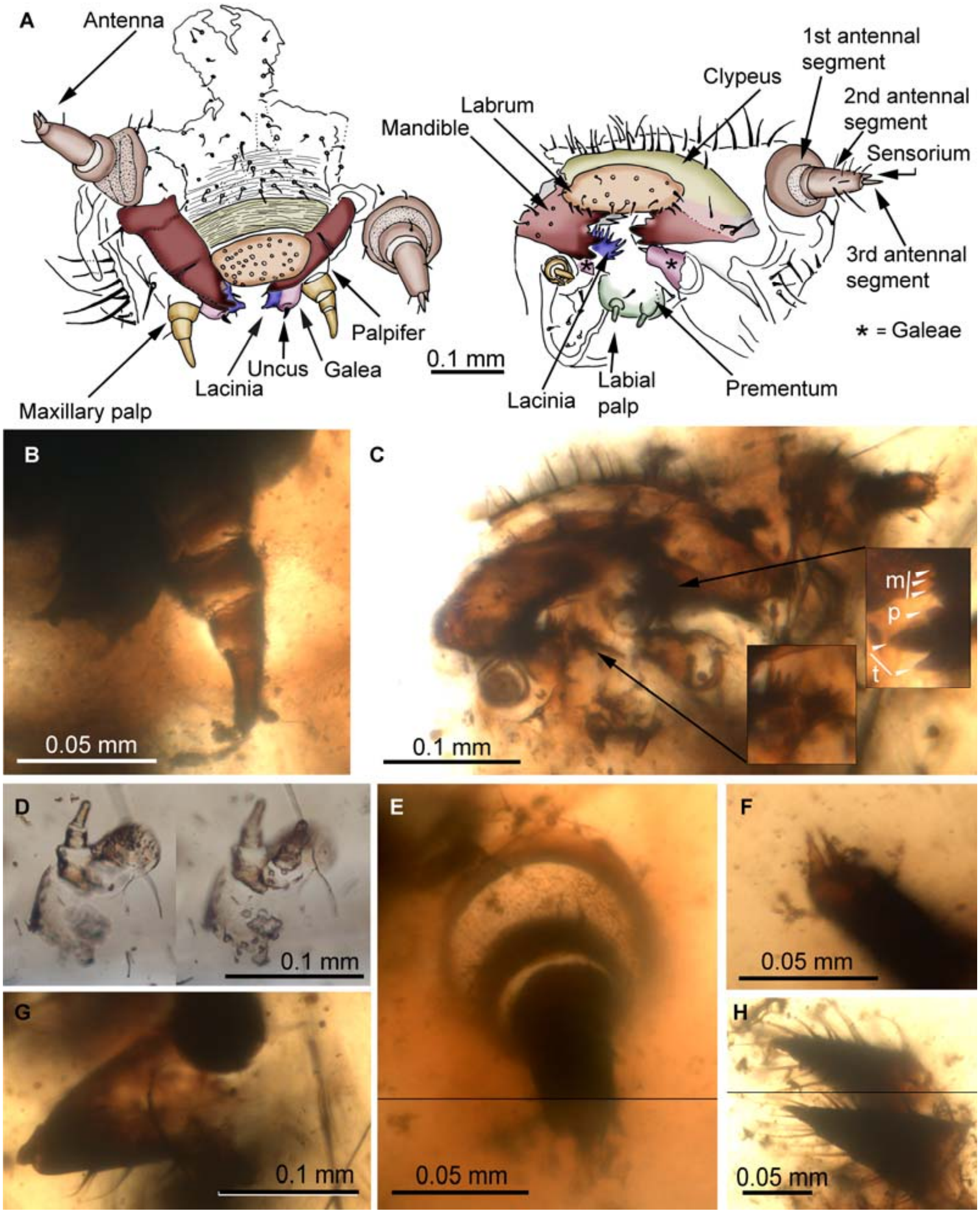

Fig. S2. Anatomical features of the exuvial remains from keratophagous beetle larvae in San Just amber, upper Albian in age. (*A*) Heads of two different specimens in frontal and ventral view, respectively. (*B*) Left maxilla showing palpus, galea and lacinia. (*C*) Head in ventral view; insets show the right lacinia (left) and left mandibular elements (right) in different focal planes. (*D*) Isolated prementum area with labial palpi, in two different focal planes. (*E* and *F*) Detail of left antenna and apex of right antenna, respectively, showing the putative flagellum (third segment) and sensorium. (*G*) Abdominal projection detached from abdominal segment VIII. (*H*) Urogomphi; note the minute spines at the apices. (*A*) (left), (B, E, F and H) from isolated exuvium (SJNB2012-11); (*A*) (right), (*C* and *G*) from almost intact exuvium among feather elements in fragment F2 (SJNB2012-31-01); (*D*) from exuvial/larval remains close to feather elements in fragment F3 (SJNB2012-31-01). Abbreviations: m = mola, p = prostheca, t = tooth. Images (*E* and *H*) composed of photographs taken at different focal planes.

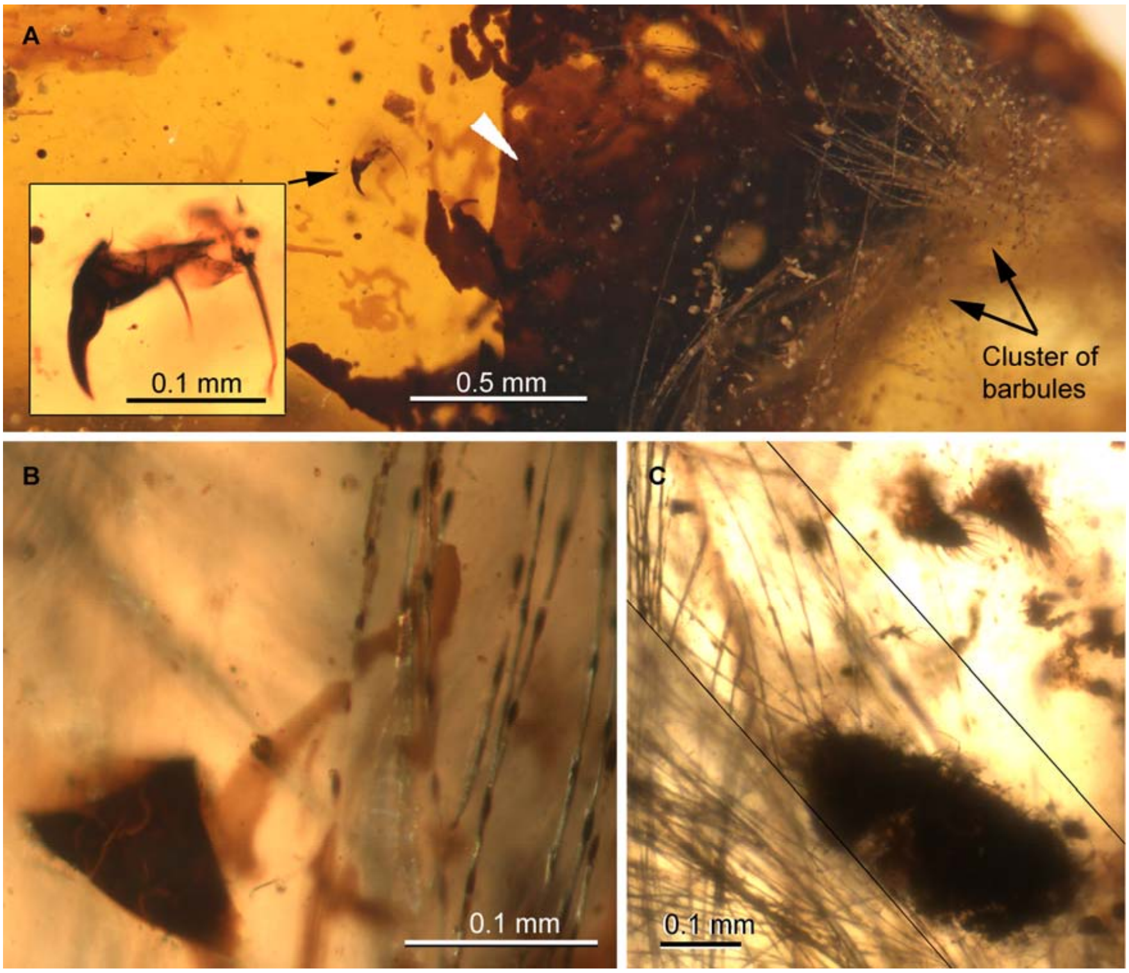

Fig. S3. Instances of fragmentary exuvial remains and fungal growth on fecal material in the preparation SJNB2012-31-01 from San Just amber, upper Albian in age. (*A*) Isolated thoracic leg apex showing tarsungulus (enlarged in inset) close to a cluster of barbules, and a dark desiccation surface typical of aerial amber (white arrowhead). (*B*) Fragment of an abdominal projection present among barbules, showing a high degree of degradation. (*C*) Fecal material (dark barrel-shaped mass) with fungal mycelia (ca. 1 μm thick) growing on its surface; this structure is present among barbules and was likely voided by the keratophagous beetle larvae (of the same or more advanced developmental stage) before molting; note the isolated, paired larval urogomphi nearby. (*A*) from amber fragment F4; (*B* and *C*) from F3 (see *SI Appendix*, Fig. S1). Image (*C*) composed of photographs taken at different focal planes.

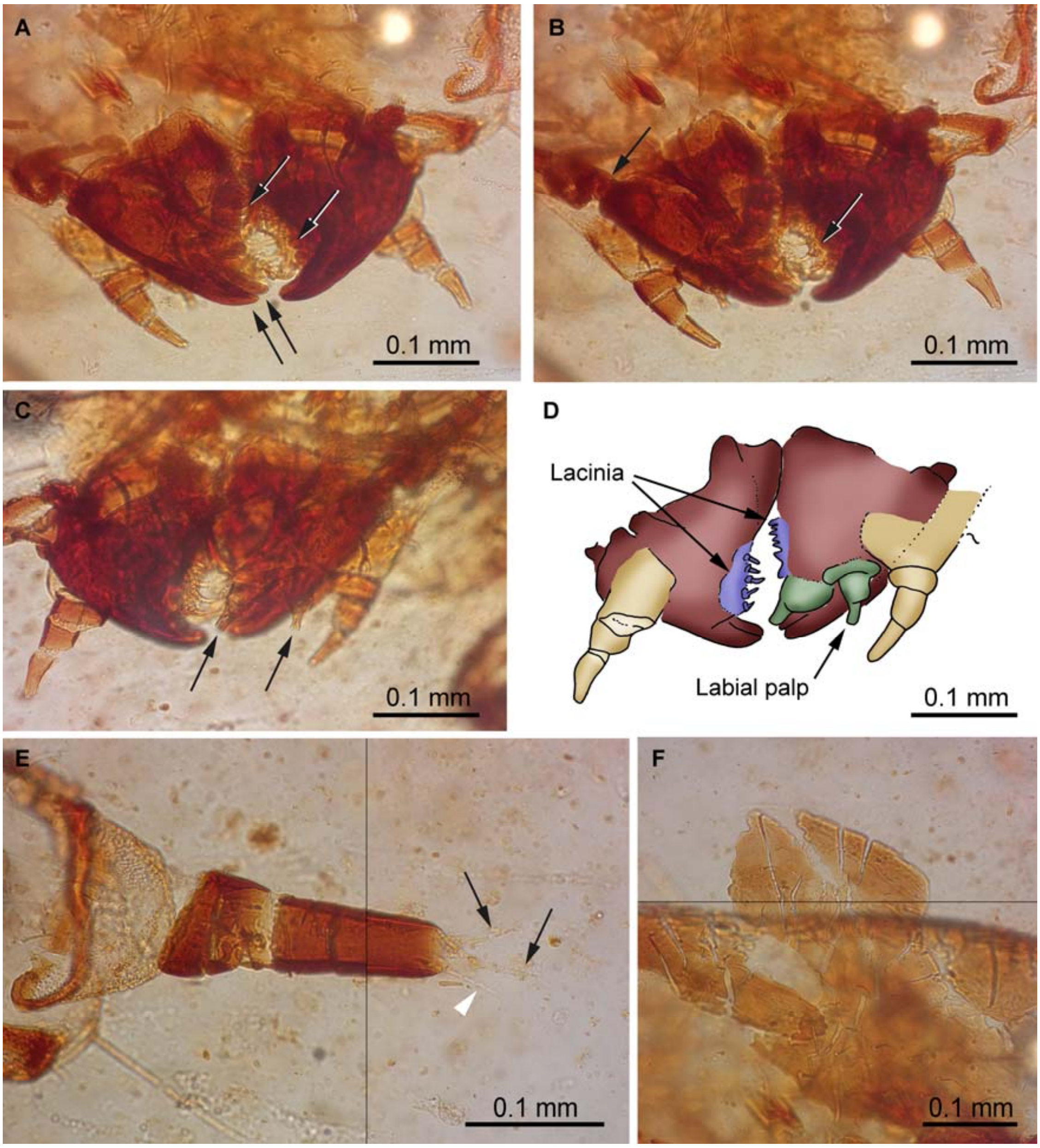

Fig. S4. Cephalic anatomical details of the isolated intact exuvium of keratophagous beetle larva from El Soplao amber, middle Albian in age, specimen ES-07-39. (*A*) Head in dorsal view showing the two subequal mandibular teeth in apical position (bottom arrows), right lacinia (top arrow) and double prosthecal process (right arrow). (*B*) Different focal plane of (*A*), slightly more ventral, showing other details of the mouthparts (left arrow indicates the mandibular condyle, right arrow indicates the left lacinia). (*C* and *D*) Head in ventral view showing other mouthparts and schematic drawing coloring the main visible parts (arrows in (*C*) indicate the two labial palps, apparent in this ventral view). (*E*) Detail of the left antenna in dorsal view (arrows indicate the sensorium –top– and the putative flagellum (third antennal segment); white arrowhead indicates the distal seta present). (*F*) Detail of the frons in dorsal view, showing lyriform frontal arms of the epicranial suture. Images (*E* and *F*) include photographs taken at different focal planes.

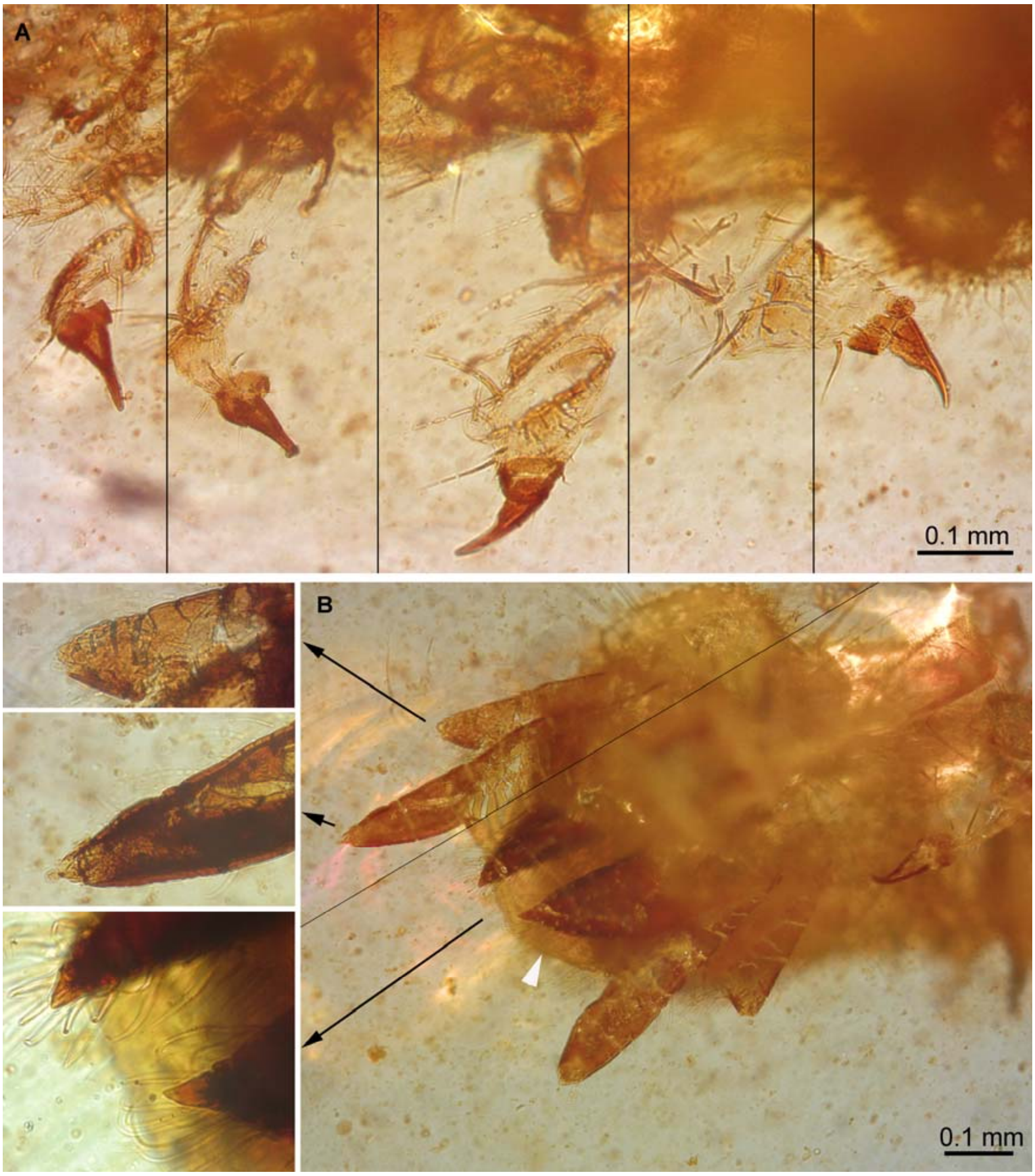

Fig. S5. Details of thoracic legs and distal abdominal structures of the isolated exuvium of keratophagous beetle larva from El Soplao amber, middle Albian in age, piece ES-07-39. (*A*) Four thoracic legs (see Fig. 3*E*) in ventral view; note their well-preserved tarsunguli. (*B*) Disposition of pair of lateral projections from abdominal segments VII and VIII and pair of urogomphi on the urogomphal plate (white arrowhead indicates the ventro-distal border of the urogomphal plate); three details of the apices of these structures from (*B*) are shown in the left insets (not to the same scale), the bottom inset shows the two apices of the urogomphi with their terminal spines. Images (*A* and *B*) include photographs taken at different focal planes.

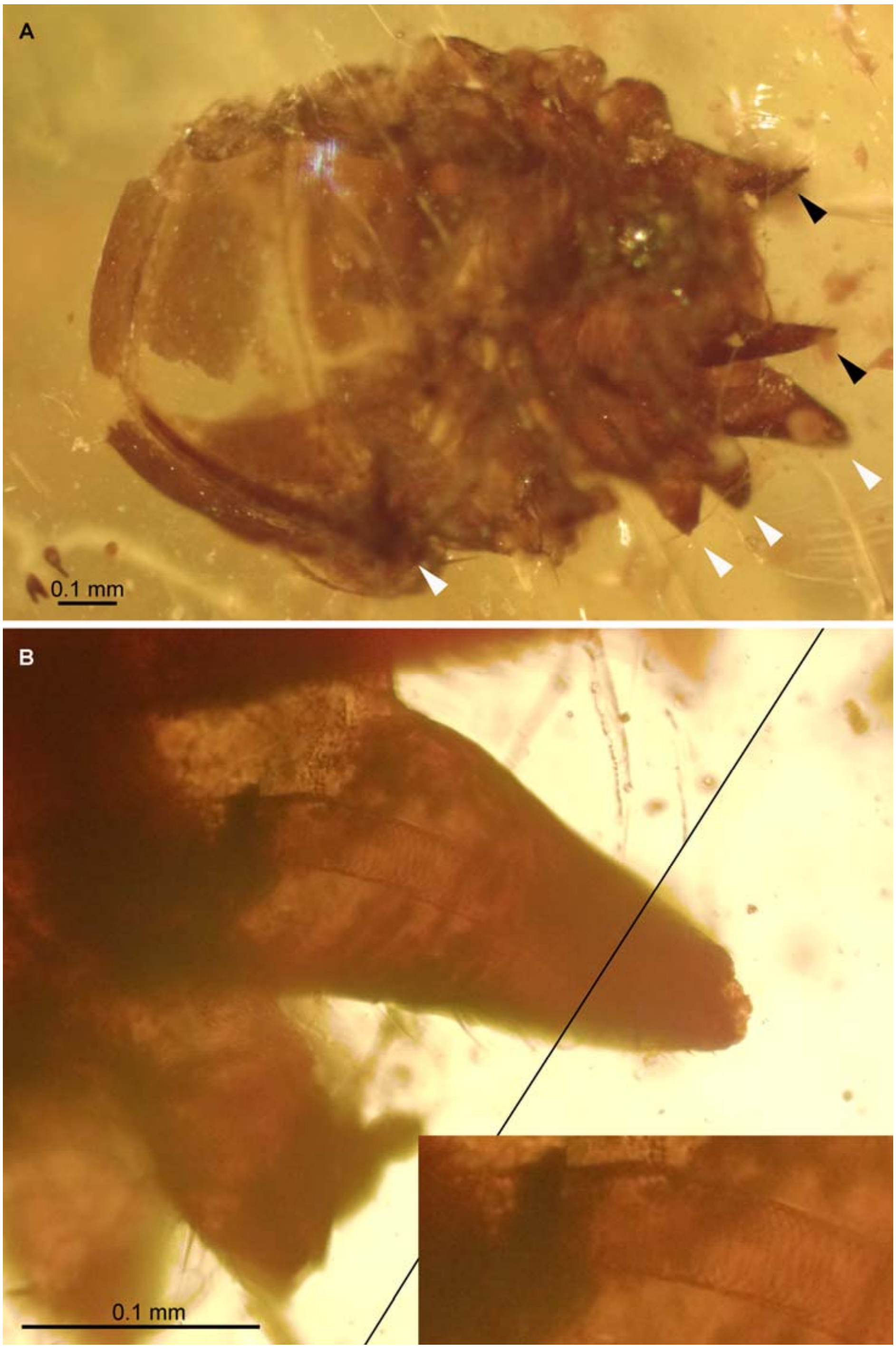

Fig. S6. Details of distal abdominal structures of the isolated partial exuvium of keratophagous beetle larva from Peñacerrada I amber, upper Albian in age, piece MCNA 12063. (*A*) Dorsal view of the posterior exuvial fragment (black arrowheads indicate the pair of urogomphi and white ones at least four observed lateral abdominal projections present in one side). (*B*) Detail of a lateral abdominal projection from segment VIII, close to the urogomphi, showing the tracheal tube contacting its apex (a section of the tracheal tube is enlarged in inset below). Image (*B*) includes photographs taken at different focal planes.

Table S1. Larval characters compared between the new fossil exuviae and polyphagan beetle families of interest. The families Dermestidae, Derodontidae and (in a lesser degree) Jacobsoniidae are the ones most closely resembling the fossils from the morphological standpoint within Bostrichiformia, Clambiformia and Staphyliniformia, respectively. The families Cryptophagidae, Cavognathidae and Tenebrionidae have been included merely as representatives of Cucujiformia and because they are found to a greater or lesser extent in extant bird nests.

	Families and studied fossil exuviae						
**Anatomic structures**	**Dermestidae**	**Derodon-tidae**	**Jacobsonii-dae**	**Cryptophagi-dae**	**Cavogna-thidae**	**Tenebrionidae**	**Fossil specimens**
Vestiture	Usually including one or more types of specialized setae*, absent in a few forms such as *Orphilus*	Simple, short setae	Fine, short setae	Fine, long setae	Simple, long, sparsely distributed setae	Usually consisting of scattered fine setae, sometimes densely hirsute or with short, stout setae	Simple, long, dense setae in thorax and abdomen. Short setae on preserved cephalic capsule
Head	Moderately to strongly declined (hypognathous)	Progna-thous	Prognathous	Prognathous	Prognathous	Prognathous or slightly declined (rarely hypognathous)	Orthogna-thous, probably slightly more declined
Frontal arms of the epicranial suture	V-shaped or U-shaped, contiguous at base	Lyriform, contiguous at bases	Lyriform, distinctly distant at bases	Lyriform, contiguous at bases	Lyriform, distant at bases	V-shaped or U-shaped, rarely lyriform, contiguous at base	Lyriform, likely contiguous at base
Antenna	Very short to moderately long	Short	Very short	Long	Long	Very short to moderately long	Relatively short
Mandible apex	Often unidentate or apically rounded; bidentate in *Orphilus*, Thorictinae and most Trinodinae; tridentate in *Dermestes*	Bidentate	Bidentate	Bidentate	Bidentate, with a preapical dorsal scissorial tooth very close to apex, which may appear tridentate	Bidentate or tridentate	Bidentate
Mandible prostheca	Diverse, some without prostheca, other with prostheca bubble-like or tuberculate	Falciform	Absent	Acute, bifid or serrate	Absent	Absent	Present, a single (early instar) or double (more advanced instar) process
Mandible mola	Usually absent, present in *Orphilus*	Well developed, inner margin crenulated	Present, finely ridged	Well-developed, surface tuberculate or asperate	Absent	Usually distinct, simple, irregularly ridged or armed with fine transverse ridges	Present, with a crenulated surface
Maxilla	Galea and lacinia separated, rounded or truncate and setose	Galea and lacinia separated, in some cases with lobes or spines	Galea and lacinia separated; galea with an attached multilobed or fringed process; lacinia with a spine-like uncus	Mala (fused galea and lacinia) with apex falciform	Mala (fused galea and lacinia) with some teeth	Mala (fused galea and lacinia)	Galea and lacinia separated. Galea bearing uncus, lacinia with two apical teeth, well sclerotized

Maxillary palp	3- or 4-segmented	3-segmented	3-segmented	3-segmented	3-segmented	3-segmented	3-segmented
Labial palp	2-segmented	2-segmented	2-segmented	1- or 2-segmented	2-segmented	Almost always 2-segmented	2-segmented
Urogomphi	Absent or present in *Dermestes* and in some Attagenini, present but short in Orphilinae	Present, short to moderately long, absent in *Laricobius*	Present, simple	Present, with dorsal and lateral tubercles at base of each	Present, moderately long	Often absent, present in some taxa of diverse subfamilies†	Present, simple, relatively short. Urogomphal plate with a pair of processes
Ecology	Primarily saprophagous of animal materials (also keratophagous) or more rarely plant materials, some predatory; commonly found in bird nests	Namely mycophagous; *Laricobius* preys on adelgids (Hemiptera: Adelgidae); not known from bird nests	Saprophagous, mycophagous or xylophagous; not known from bird nests	Very diverse, although primarily mycophagous; some found in bird nests	Regarded as bird parasites but biology rather unknown, likely saprophagous; exclusively known from bird nests	Diverse, namely xylophagous or mycophagous, predaceous (particularly larvae) and saprophagous (including keratophagous) forms also present; *Ulomoides* is commonly found in bird nests	Most likely keratophagous and associated to theropod nests based on amber taphonomy
**Data sources**	(1–4)	(2, 5, 6)	(2, 7)	(2, 5)	(2)	(2)	This work
**Cretaceous amber record**	One species from Lebanese amber, eight from Burmese amber (one as larva), and one from New Jersey amber. Undescribed: larvae (8, 9); four adults from Lebanese amber (10) and nine from Spanish amber (11). Additional evidence as hastisetae (12, 13)	None	Two species in Burmese amber. One species in French amber.	One species in Lebanese amber, one species in Spanish amber, four species in Taimyr amber. Four undescribed specimens present in Spanish amber (11)	None	One species in Burmese amber. Two undescribed specimens in Spanish amber (11)	This work

* Barbed spicisetae in *Dermestes*, Trinodinae and Megatominae; ribbed, scale-like setae in Attageninae; hastisetae with apical recurved barbs in Megatominae and some Trinodini; and expanded setae in Thorictinae.
† Lagriinae, Pimeliinae, Tenebrioninae, Alleculinae, Diaperinae and Stenochiinae.

Movie S1 (separate file). Confocal laser scanning microscopy images showing a beetle exuvium among feather portions and optical microscopy images of the head from a conspecific isolated exuvium from the same stratigraphic level in upper Albian San Just amber outcrop. Preparation SJNB2012-31-01 (amber fragment F2) and piece SJNB2012-11. Both specimens viewed in frontal view. Stacking slideshows are based on photographs taken at successive focal planes.

SI References

1. T. Kiselyova, J. V. McHugh, A phylogenetic study of Dermestidae (Coleoptera) based on larval morphology. *Syst. Entomol.* **31**, 469–507 (2006).
2. R. A. B. Leschen, B. G. Beutel, J. F. Lawrence, *Handbook of Zoology. Arthropoda: Insecta. Coleoptera, beetles. Volume 2: Morphology and Systematics (Elateroidea, Bostrichiformia, Cucujiformia partim* (Walter de Gruyter, Berlin, 2010).
3. R. S. Jr. Beal, Review of Nearctic species of *Orphilus* (Coleoptera: Dermestidae) with description of the larva of *O. subnitidus* LeConte. *Coleopt. Bull*. **39**, 265–271 (1985).
4. R. D. Zhantiev, Palaearctic dermestid beetles of the genus *Orphilus* Er. (Coleoptera, Dermestidae). *Entomol. Rev.* **81**, 200–210 (2001).
5. J. F. Lawrence, “Order Coleoptera” in *Immature Insects*, F. W. Stehr, Ed. (Kendall/Hunt Publishing Company, 1991), pp. 144–658.
6. G. M. G. Zilahi-Balogh, L. M. Humble, L. T. Kok, S. M. Salom, Morphology of *Laricobius nigrinus* (Coleoptera: Derodontidae), a predator of the hemlock wooly adelgid. *Can. Entomol*. **138**, 595–601 (2006).
7. R. A. Crowson, Studies on the Dermestoidea (Coleoptera), with special reference to the New Zealand fauna. *Trans. R. Entomol. Soc. Lond.* **111**, 81–94 (1959).
8. D. A. Grimaldi, M. S. Engel, *Evolution of the Insects* (Cambridge University Press, Cambridge, 2005).
9. D. Peris, J. Rust, Cretaceous beetles (Insecta: Coleoptera) in amber: the palaeoecology of this most diverse group of insects. *Zool. J. Linn. Soc.* zlz118 (2019). doi.org/10.1093/zoolinnean/zlz118
10. A. G. Kirejtshuk, D. Azar, P. Tafforeau, R. Boistel, V. Fernandez, New beetles of Polyphaga (Coleoptera, Polyphaga) from Lower Cretaceous Lebanese amber. *Denisia* **26**, 119–130 (2009).
11. D. Peris, E. Ruzzier, V. Perrichot, X. Delclòs, Evolutionary and paleobiological implications of Coleoptera (Insecta) from Tethyan-influenced Cretaceous ambers. *Geosci. Front.* **7**, 695–706 (2016).
12. E. Peñalver, A. Arillo, X. Delclòs, D. Peris, D. A. Grimaldi, S. R. Anderson, P. C. Nascimbene, R. Pérez-de la Fuente, Ticks parasitised feathered dinosaurs as revealed by Cretaceous amber assemblages. *Nat. Commun.* **8**, 1924 (2017). doi.org/10.1038/s41467-017-01550-z
13. G. O. Jr. Poinar, R. Poinar, Ancient hastisetae of Cretaceous carrion beetles (Coleoptera: Dermestidae) in Myanmar amber. *Arthropod Struct. Dev.* **45**, 642–645 (2016).
